# Radiant Energy Not Curative of Cancer

**Published:** 1914-05

**Authors:** 


**Radiant Energy Not Curative of Cancer.** Czerny of Berlin, with an experience of 4,000 cases since 1906, holds that radium and mesothorium have practically identical action, that even X-rays do not penetrate more than 4—5 c.m. to have a destructive action, and that while superficial cells are killed by any of these forms of radio-activity, the deeper cells are stimulated.

